# Psychiatrists’ Readiness for Digital Psychiatry in Pakistan: A Multicenter CrossSectional Study with Regression Analysis

**DOI:** 10.1192/j.eurpsy.2024.1138

**Published:** 2024-08-27

**Authors:** M. M. Adeel Riaz, T. Abrar, S. S. Tebha, H. Tariq, R. Fatima, M. Hamad, A. A. Khan, A. Ahadi, N. Ul Sabah, W. Rasool, K. Latif, M. Umar, I. Siddique, F. A. Nawaz

**Affiliations:** ^1^Punjab Medical College, Faisalabad; ^2^Khyber Medical College, Peshawar; ^3^Department of Neurosurgery and Neurology, Jinnah Medical and Dental College; ^4^SHINE Humanity, Karachi; ^5^Faisalabad Medical University, Faisalabad; ^6^Riphah School of Business and Management, Riphah International University; ^7^Shifa College of Pharmaceutical Sciences, Shifa Tameer-e-Millat University; ^8^Shifa College of Medicine, Shifa Tameer-e-Millat University, Islamabad; ^9^Ayub Medical College, Abbotabad; ^10^Bolan Medical College, Quetta; ^11^Quaid-e-Azam Medical College, Bahawalpur; ^12^ King Edward Medical University; ^13^Fatima Jinnah Medical University, Lahore; ^14^Faculty of medicine, Khairpur Medical College, Khairpur mirs, Sindh; ^15^Department of Psychiatry and Behavioural Sciences, Faisalabad Medical University, Faisalabad, Pakistan; ^16^Emirates Health Services, Al Amal Psychiatric Hospital, Dubai, United Arab Emirates

## Abstract

**Introduction:**

The concept of digital psychiatry, encompassing technologies such as mental health apps, Virtual Reality (VR), Artificial Intelligence (AI), and telepsychiatry, emerges as a potential solution to bridge the existing gaps in the mental health system of Pakistan. However, one of the major barriers to the implementation of these technologies is hesitancy to adopt digital tools by psychiatrists.

**Objectives:**

This study aims to explore the current understanding of digital psychiatry, the barriers faced by psychiatrists in its’ widespread implementation, and their willingness to adopt these services in clinical practice.

**Methods:**

This cross-sectional study surveyed psychiatrists’ knowledge, attitudes, and practices on digital psychiatry from 39 public hospitals across Pakistan using an online validated questionnaire from January to July 2023. Participants included psychiatry residents, fellows, and consultants practicing in Pakistan. Responses were analyzed with Raosoft software, Quirkos, and SPSS 26 using thematic analysis and correlation.

**Results:**

A total of 200 participants responded to the questionnaire, primarily in the age range of 20-30 years (56%). The gender distribution was 55% male (N = 111) and 45% female (N = 89). Among the professional roles, 23% were consultants, 7% were registrars, 54% were psychiatry residents, and 17% were medical officers. Respondents came from both rural (N = 148, 74%) and urban (N = 52, 26%) practice settings. Regarding telepsychiatry, 46% strongly agreed that they are familiar with telepsychiatry, while 58% agreed that telepsychiatry can save time and money. Additionally, 22% strongly agreed that it’s a viable approach for patient care. Concerning perspectives on Artificial Intelligence (AI) in digital psychiatry readiness, only 40% of participants had received AI training. However, 55% expressed interest in collaborating with international centers on AI-related projects. In terms of mental health apps, 62% of respondents reported limited familiarity with them. Nevertheless, 65% believed that these apps could potentially save time and money for psychiatric health systems. Lastly, concerning Virtual Reality (VR) in psychiatric care, 57% of participants were familiar with VR technology, but only 43% were acquainted with its applications in psychiatry. Notably, 71% did not view VR as a viable replacement for in-person psychiatric management.

**Conclusions:**

This is the first study conducted on understanding digital psychiatry in Pakistan’s healthcare system, which revealed multiple challenges to digital health competency among psychiatrists. This emphasizes on the need for formal training and funding towards resources to overcome obstacles in utilizing mental health technologies.

**Disclosure of Interest:**

None Declared

